# App-based telerehabilitation program for older adults on waiting list for physiotherapy after hospital discharge: a feasibility pragmatic randomized trial

**DOI:** 10.1186/s40814-024-01521-4

**Published:** 2024-07-03

**Authors:** Pollyana Ruggio Tristão Borges, Rosana Ferreira Sampaio, Jane Fonseca Dias, Marisa Cotta Mancini, Juliana Melo Ocarino, Renan Alves Resende

**Affiliations:** Department of Physiotherapy, Graduate Program in Rehabilitation Sciences, Universidade Federal de Minas Gerais, Avenida Antônio Carlos, 6627 - Campus Pampulha, Belo Horizonte, Minas Gerais 31270-901 Brazil

**Keywords:** Telehealth, Deconditioning, Rehabilitation, Geriatrics, Hospitalization

## Abstract

**Background:**

Inactivity while waiting for outpatient physiotherapy worsens the physical deconditioning of older adults after hospital discharge. Exercise programs can minimize the progression of deconditioning. In developing countries, telerehabilitation for older adults on the waiting list is still in the early stages. This study aimed to evaluate the feasibility of the study procedures of a telerehabilitation program for older adults waiting for outpatient physiotherapy after hospital discharge.

**Methods:**

This pragmatic randomized controlled trial recruited older adults (≥ 60 years) with several clinical diagnoses on the waiting list for outpatient physiotherapy in the Brazilian public health system after hospital discharge. The telerehabilitation group (*n* = 17) received a personalized program of multicomponent remote exercises using a smartphone app. The control group (*n* = 17) followed the usual waiting list. We assessed recruitment and dropout rates, safety, adherence, and satisfaction. The preliminary effects were verified on clinical outcomes.

**Results:**

We recruited 5.6 older adults monthly; dropouts were 12%. No serious adverse events were associated with the telerehabilitation program. The weekly adherence was 2.85 (1.43) days, and in 63.3% of the weeks the participants were enrolled, they performed the exercise program at least twice a week. Participants rated the telerehabilitation program as 9.71 (0.21), and the safety of remote exercises without professional supervision as 8.6 (2.2) on a 0–10 scale.

**Conclusions:**

The telerehabilitation program using a smartphone app was safe and presented high participants’ satisfaction and adequate adherence, recruitment, and dropout rates. Therefore, the definitive study can be conducted with few modifications.

**Trial registration:**

Brazilian Registry of Clinical Trials (ReBEC), RBR-9243v7. Registered on 24 August 2020. https://ensaiosclinicos.gov.br/rg/RBR-9243v7.

**Supplementary Information:**

The online version contains supplementary material available at 10.1186/s40814-024-01521-4.

## Key messages regarding feasibility

- What uncertainties existed regarding the feasibility?
  A comprehensive investigation is needed to evaluate the feasibility of implementing a telerehabilitation program for older adults post-hospital discharge who are vulnerable to health issues, possess lower educational levels, and lack experience with technology.
- What are the key feasibility findings?
  Recruitment and dropout rates, safety, adherence, and satisfaction with the proposed intervention met a priori thresholds for feasibility.
- What are the implications of the feasibility findings for the design of the main study?
  The findings of this study provide information about the feasibility of the telerehabilitation program to better guide the definitive study, such as no need of companion for independent older adults during exercise.

## Background

Older adults on the waiting list for outpatient physiotherapy after hospital discharge are susceptible to deconditioning due to the negative effects of inactivity during hospitalization and after discharge. Deconditioning is a systemic physiological change [1, 2] associated with increased mortality, hospital readmissions, and institutionalization [3, 4]. Deconditioning may initiate during hospitalization [5] and continue months after discharge [6, 7]. In this sense, long waiting lists for rehabilitation increase cost for the health system and dissatisfaction among patients and families, impairing the recovery of patients [8–10]. The treatment of deconditioned older adults must involve a multicomponent intervention based on high-intensity resistance exercises, balance training, and activities of daily living [2]. Thus, exercise programs during the waiting period may minimize the progression of deconditioning in older adults caused by inactivity and delayed rehabilitation.

Telerehabilitation is an emerging physiotherapy modality widely used during social isolation due to the COVID-19 pandemic [11, 12]. This modality provides treatment or orientation through communication technologies such as smartphone apps [12]. Also, telerehabilitation had superior or equal effectiveness to in-person physiotherapy for patients with different musculoskeletal conditions [13–16]. Remote rehabilitation is safe [14, 17], has good acceptability and satisfaction for most patients [18, 19], and reduces costs of patients and health care services [20–22]. As the smartphone is the communication device most used to access the internet [23, 24], it can also be used for remote treatment [14, 25] and delivery of exercise programs [26, 27]. Nevertheless, remote rehabilitation using technology may not be feasible for all countries, especially developing countries [15, 28].

In this context, a telerehabilitation program using a smartphone app was developed to minimize deconditioning of older adults waiting for outpatient physiotherapy in the public health system after hospital discharge [29]. A pragmatic randomized clinical trial protocol was designed to verify the effectiveness of this program. However, a feasibility study is needed before implementing the main study, since older adults after hospital discharge have multiple comorbidities, different clinical diagnoses, low educational levels [30], and inexperience with technology management [31]. This feasibility study will support the decision-making regarding the continuity of recruitment and allow adjustments in the original protocol to reduce time and costs [32, 33]. This study aimed to (1) evaluate the feasibility of the study procedures of a multicomponent telerehabilitation program using a smartphone app for older adults on the waiting list for outpatient physiotherapy after hospital discharge for any specific medical condition and (2) to determine if the study should continue (with or without modifications).

## Methods

### Trial design

This is a feasibility study of a pragmatic randomized controlled clinical trial with two parallel arms, not conducted independently of the main trial. The clinical trial protocol has been published [29] and prospectively registered at Brazilian Registry of Clinical Trials, RBR-9243v7. This study followed the Consolidated Standards of Reporting Trials extension for randomized pilot and feasibility trials [34], as detailed in Additional file 1.

### Setting and participants

**Fig. 1:**
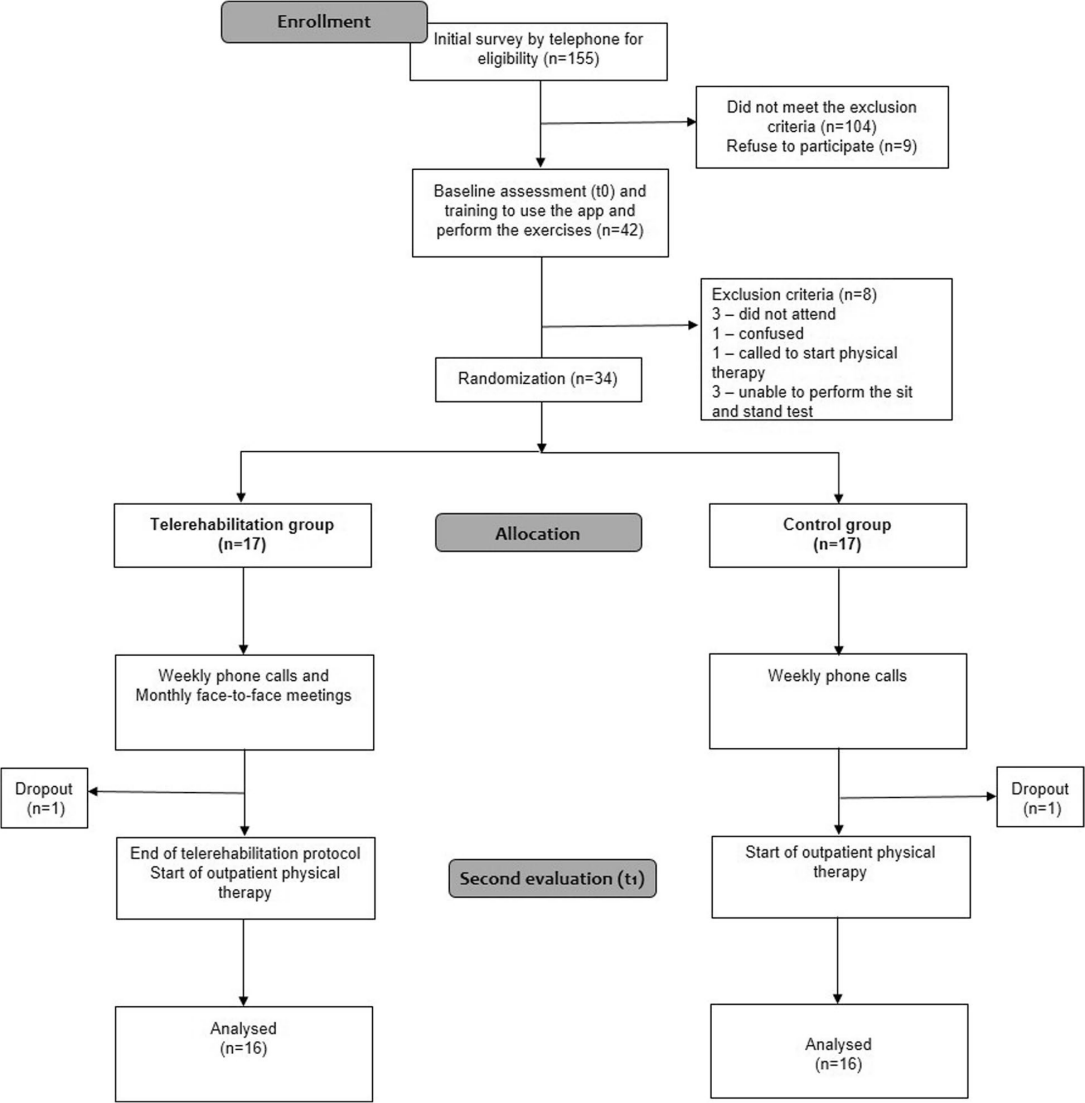
Study flowchart

Thirty-four older adults were recruited from the waiting list for outpatient physiotherapy in the public health system of Belo Horizonte (Brazil) between May and September 2021. Researchers contacted potentially eligible older adults and invited those who met the inclusion criteria to an in-person evaluation. Figure 1 details the study flowchart. All participants signed an informed consent form.

The following participants were included: age above 60 years; referred to outpatient physiotherapy in the public health system after hospital discharge; no restriction regarding lower limb weight bearing; independent walking (with or without assistance device); able to sit and stand from a chair and understand oral instructions and commands during tests; clinical stability; have a smartphone with internet connection (own or companion) and a companion while performing the remote exercises. Older adults with clinical complications hindering physical exercise, neurological diseases, and a score on the Mini-Mental State Examination (0–30) below the cut-off point according to educational level—13 for illiterate, 18 for elementary and middle, and 26 for high educational level—were excluded [35].

### Study procedures

Participants were randomized in blocks into control (CG) and telerehabilitation (TG) groups using a 1:1 allocation ratio. The allocation was in random blocks sizing four and six. Before data collection, a person not involved in the study confidentially generated the randomization list [36] and placed it in opaque, sealed, and sequentially numbered envelopes.

**Fig. 2:**
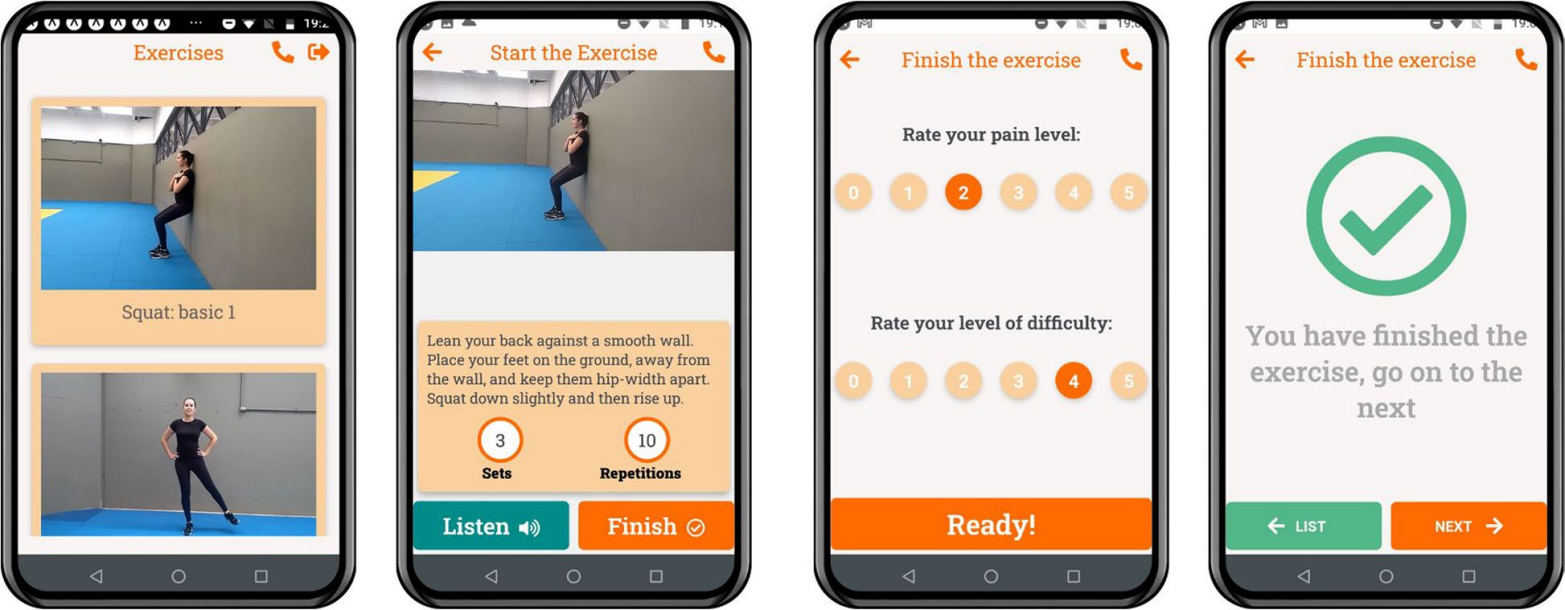
Telefisio app

The TG received a personalized program of multicomponent, global, and simple remote exercises (Table 1). A trained physical therapist assessed the participants to identify their functional ability and determine the exercises’ difficulty levels and their sublevels. It was considered the correct execution of the exercise, safety, participant’s effort, and discomfort, always opting for the most appropriate level for the older adult’s functional ability. Exercise intensity was based on Borg’s Modified Perceived Exertion Scale (0–10), which should range between 5 and 7. All participants were instructed to perform exercises at home in the presence of a companion. Exercises were available in the *Telefisio* app (Fig. 2) for smartphones, developed for this study (available at https://play.google.com/store/apps/details?id=com.telefisio). Participants in the TG were contacted weekly by phone call to encourage adherence, monitor the performance and adverse events of the program, and identify other interventions. These individuals were monitored in-person every 25 to 30 days at the participant’s rehabilitation center. The full detailed telerehabilitation program is presented in the clinical trial protocol [29].

**Table 1:** Overall exercise program view

Exercise	Description	Possible progressions
Multiplanar single-leg balance reach	The participant starts in the standing position, assumes single lower limb support, and moves the opposite lower limb forward, to the side, and backward as far as possible and maintains the knee as straight as possible	Increase number of repetitions Increase swing limb reach distance Remove upper limb support Restrict touch on the floor with the forefoot
Squat	The participant starts this exercise in the standing position with the back supported by a wall and then squat	Increase number of repetitions Increase range of motion Remove back support but allow bilateral upper limb support Allow unilateral upper limb support Simulating sitting on a chair
Forward lunge	The participant starts this exercise in the standing position and then performs a forward lunge alternating lower limbs	Increase number of repetitions Increase range of motion Remove upper limb support Simultaneous trunk rotation
Lateral lunge	The participant starts this exercise in the standing position and then performs lateral lunge alternating lower limbs	Increase number of repetitions Increase range of motion Remove upper limb support Simultaneous trunk rotation
Pushup	The participant starts this exercise in the standing position with feet shoulder-width apart and facing a wall free from any objects or obstacles. The participant is just over one arm’s length away from the wall. Then, the participant puts the palms of both hands flat against the wall at shoulder height, approximately shoulder-width apart, and then lower and lift him/herself against the wall while keeping their feet planted firmly on the floor and maintaining the back and hips straight. The progression to advanced level includes kneeling and floor pushup	Increase number of repetitions Increase range of motion Kneeling pushup Floor pushup
Daily activity	The participant chooses an activity from their daily routine that they have difficulty in performing and hope to improve in the short term

The CG did not receive any intervention or instruction; only weekly phone calls were made to monitor co-interventions and adverse events.

The time of participation in this study depended on how long participants remained on the waiting list for outpatient physiotherapy.

### Primary outcome measures

#### Recruitment rate

The number of participants recruited during the study was divided by the number of months of data collection.

#### Dropout rate

The dropout rate is the number of participants who dropped out of the study.

#### Safety of the telerehabilitation program

Participants were asked about adverse events related to the exercise program (e.g., fatigue, dizziness, disabling pain, and falling) during weekly phone calls. Muscle pain and fatigue were considered non-serious events. Participants were also asked about events unrelated to the intervention. At the end of the program, participants rated the perceived safety of performing exercises at home on a 0–10 scale.

#### Adherence to the telerehabilitation program

Exercise performance was monitored through access and time of use of the Telefisio app and during weekly phone calls to the TG. Adherence was measured considering the mean number of days per week that the participant performed the program during waiting time. In addition, following the World Health Organization recommendations [37] and considering the adherence challenges faced by older adults after hospital discharge, adherence was assessed as the percentage of weeks that the participants performed the program at least twice a week.

#### Satisfaction with the telerehabilitation program

Participants rated their overall satisfaction with the telerehabilitation program on a 0–10 scale. They also reported the probability of future participation and recommendation of the program to family or friends on a 0–10 scale.

### Secondary outcome measures

Secondary outcomes were measured at baseline and start of the outpatient physiotherapy (t1); outcomes assessors were blinded to group allocation.

#### Timed Up and Go (TUG)

The time for the participant to stand from a chair, walk 3 m, and return to sit in the same chair was counted. This test was developed to measure the functional mobility of older adults [38]. Studies have shown that this test has a Minimum Clinically Important Difference of 0.8 s [39, 40].

#### 30-s Chair Stand Test (30CST)

The number of times the participant could stand and sit down from a chair as fast as possible for 30 s was included for analysis. The 30CST was developed as a measure of lower limb strength for older adults and has excellent reliability (*r* = 0.89) [41].

#### Pain

Pain level was assessed using a numerical visual analog scale (0–10) graded in colors. This instrument is valid and reliable for the older adult population (ICC = 0.84) [42].

#### Physical health

Physical health was measured using the Physical Functioning Scale of the Brazilian version of the Medical Outcomes Short-Form Health Survey. This version has established validity and reliability (ICC > 0.80) [43]. The final score of the scale ranges from 10 to 30, and low scores reflect increased perceived limitations.

#### Health-related quality of life

General health status was measured using the visual analog scale (0–100) of the EuroQol-5D-3L instrument. Values of 100 represented “the best health you can imagine” and 0 “the worst health you can imagine.” The EQ-5D-3L have proven to be reliable (ICC = 0.52–0.83) [44].

#### Perceived performance and satisfaction with an important activity

We used the Canadian Occupational Performance Measure, which has been tested for measurement properties, including reliability (ICC = 0.67–0.69) [45]. Participants chose a self-care activity considered the most important and scored their performance and satisfaction with this activity on a 1–10 scale. Low scores indicate reduced performance and satisfaction.

### Criteria to proceed with the definitive study

The criteria defined to proceed with the definitive study were based on previous literature: monthly recruitment rate of at least four participants, dropout rate less than 15% [46], no serious adverse events associated with the telerehabilitation program [47], mean adherence of at least twice a week [37], and satisfaction score with the program of at least six [48].

### Sample size

Following established guidelines that recommend a minimum of 20 participants for feasibility studies [49, 50], we included 34 older adults in this study. With this simple size, and presuming an 85% retention rate, we were able to estimate retention to a margin of approximately 13%, with a confidence level of 95%.

### Statistical analysis

Descriptive statistics were used to analyze the feasibility outcomes: range and mean for recruitment rate; percentage for dropout rate; mean and 95% confidence intervals (CI) for safety of the telerehabilitation program, and satisfaction with the telerehabilitation program; and percentage, mean, and 95% CI for adherence to the telerehabilitation program. Sample characteristics at baseline and secondary outcomes were descriptively analyzed. The mean within-group differences between baseline and start of physiotherapy were calculated, and the mean differences between groups with 95% confidence intervals (CI) were reported for the difference of these means. In addition, we conducted a priori power analysis for ANCOVA, setting parameters at 80% power, an alpha level of 0.05, and a 6% dropout rate to refine the sample size calculation for the definitive trial using G*Power software (Additional file 2). The effect sizes for TUG were derived from partial eta squared (η2) values in the ANCOVA. We used the delta values of TUG (difference from the start of outpatient physiotherapy to baseline) as dependent variables, with waiting time for outpatient physiotherapy and baseline value as covariates, and group as independent variable, which resulted in an effect size of 0.17. All analyses considered the intention-to-treat approach by including participants who did not adhere to the telerehabilitation program.

## Results

### Sample characteristics

The main characteristics of the sample can be found in Table 2. In summary, the mean age of the sample was 66.5 years (SD = 5.1), and the majority of the sample was female (61.8%). Regarding education, the lowest level of education was 0 and the highest was 16 years. Approximately 74% of participants had at least one comorbidity, and 62% reported at least one fall during the last year. Participants remained hospitalized for 1 to 45 days, mostly due to musculoskeletal diseases in the lower limbs that resulted in surgeries (44.1%).

**Table 2:** Sample characteristics

	**Telerehabilitation group (*N* = 17)**	**Control group (*N* = 17)**	**Total (*N* = 34)**
**Age (years), mean (SD)**	65.6 (5.3)	67.3 (5.0)	66.5 (5.1)
**Gender (*n*,%)**			
Female	9 (52.9)	12 (70.6)	21 (61.8)
Male	8 (47.1)	5 (29.4)	13 (38.2)
**Marital status, *n* (%)**			
Married or with a partner	9 (53)	9 (53)	18 (52)
Single	8 (47)	8 (47)	16 (50)
**Formal education, *n* (%)**			
Elementary and middle﻿	10 (58.8)	11 (64.7)	21 (61.8)
High	5 (29.4)	5 (29.4)	10 (29.4)
Illiterate	2 (11.8)	1 (5.9)	3 (8.8)
**Comorbidities, *n* (%)**			
None	6 (35.3)	3 (17.6)	9 (26.5)
At least one	6 (35.3)	8 (47.1)	14 (41.1)
Two or more	5 (29.4)	6 (35.3)	11 (32.4)
**Falls, *n* (%)**			
None	7 (41.2)	6 (35.3)	13 (38.2)
At least one	10 (58.8)	11 (64.7)	21 (61.8)
**Length of hospital stay (days), mean (SD)**	9.8 (11.6)	11.3 (11.2)	10.6 (11.3)
**Disease, *n* (%)**			
Musculoskeletal in the lower limbs	9 (53)	7 (41.2)	16 (50)
Musculoskeletal in the upper limbs﻿	2 (11.8)	4 (23.6)	6 (17.6)
Spine	3 (17.6)	3 (17.6)	6 (17.6)
Respiratory	2 (11.8)	3 (17.6)	5 (11.9)
Vascular diseases	1 (5.8)	0 (0.0)	1 (2.9)
**Waiting time (days), mean (SD)**	53 (37.1)	21.4 (21.1)	36.7 (33.6)

### Primary outcome measures

#### Recruitment rate

A total of 155 older adults on the waiting list for outpatient physiotherapy were potentially eligible for the study and contacted. Of these, 104 did not meet one of the inclusion criteria, and nine refused to participate due to different reasons (i.e., lack of money, and preference for in-person treatment at the rehabilitation center even with the long waiting period). Forty-two older adults were scheduled for the initial in-person evaluation; eight were excluded before randomization (Fig. 1). The monthly number of randomized participants ranged from 3 to 10, and the monthly recruitment rate was 5.6 participants.

#### Dropout rate

Two participants (6%) dropped out of the study, one in TG and one in CG. In TG, the participant did not answer weekly phone calls and we lost communication. In the CG, the participant dropped out for considering the weekly monitoring unnecessary. Two other participants in the TG stopped participation in the intervention—excessive pain unrelated to exercise, other health problems unrelated to hospitalization, internet failure, and discouragement—but opted for revaluation at the beginning of the outpatient physiotherapy. Consequently, there were 16 participants with complete data for secondary outcome analysis in both groups.

#### Safety of the telerehabilitation program

No serious adverse events were associated with the telerehabilitation program; only fatigue and pain in the lower limbs after exercises were reported. Events unrelated to the program included gallbladder pain, infection in the surgical site, fall, hemorrhoids, flu, and bowel pain. These events stopped the complete weekly performance of exercises during the program. In the CG, inflammation in the surgical site and pain were reported. No unintended consequences or harms were reported.

The mean score for the safety of remote exercises without professional supervision was 8.6 (SD = 2.2; 95% CI = 7.4 to 9.8). One participant reported a score lower than 7, demonstrating insecurity in performing the remote exercises.

#### Adherence to the telerehabilitation program

The participants performed the exercises 2.8 days per week (SD = 1.4; 95% CI = 1.0 to 6.0). In 63.3% (95% CI = 40 to 80%) of the weeks they were enrolled, participants performed the exercise program at least twice a week. Of the 14 participants who completed the telerehabilitation program, 10 performed the exercises at least twice per week and for at least 69% (95% CI = 40 to 90%) of the waiting period. Additionally, of these participants, 50% (7/14) used the smartphone app at least twice a week throughout the entire study. One participant did not perform the exercises twice per week during the telerehabilitation period. However, this participant’s waiting period for outpatient physiotherapy was only 2 weeks.

The reasons for non-adherence to the telerehabilitation program included health problems unrelated to the program (e.g., infection in the surgical site and falls), lack of time, unavailability of a companion, and lack of internet. Most participants with low adherence to the program reported lack of time and unavailability of a companion. Four participants had lack of internet service; two of them performed the exercises and registered in the app when the internet was available.

Six participants performed exercises alone (even with instructions requesting a companion) since they had a smartphone and were familiar with its use.

Only one participant did not use the app due to lack of internal storage capacity in the smartphone, and the companion refused to install it on another device. However, this participant memorized and performed all exercises (self-report adherence).

#### Satisfaction with the telerehabilitation program

Fourteen participants from the TG responded about satisfaction with the telerehabilitation program. The mean general satisfaction score was 9.7 (SD = 0.6; 95% CI = 8.0 to 10.0). Almost all participants would recommend the program to family or friends (mean score of 9.9; SD = 0.2; 95% CI = 9.0 to 10.0). The mean score for future participation was 9.5 (SD = 1.4; 95% CI = 5.0 to 10.0). Reports about participation included satisfaction regarding attention and interaction with researchers, innovation of performing remote exercises using a smartphone app, opportunity to learn how to use the smartphone, and family fun while performing exercises at home.

### Secondary outcome measures

Participants in the CG spent 21.4 days (SD = 21.1) in the study, whereas those in the TG spent 53 days (SD = 37.1). Clinical measures of both groups at baseline and start of outpatient physiotherapy are shown in Table 3. Preliminary results indicated the non-progression of deconditioning in the TG and also in the CG (Table 3). Both the TG and CG groups showed improvements in the TUG test and the perceived performance and satisfaction in COPM.

**Table 3:** Clinical measures in both groups at baseline and start of outpatient physiotherapy

	**Telerehabilitation group**		**Control group**		**Mean between groups difference (95% CI)**
	**Baseline *n* = 17**	**t1 *n* = 16**	**Baseline *n* = 17**	**t1 *n* = 16**	**Telerehabilitation minus control**
**TUG (seconds)**	16.3 (10.5)	13.5 (7.0)	14.2 (6.9)	13.3 (6.9)	− 1.9 (− 5.2 to 1.4)
**30CST (repetitions)**	8.5 (2.0)	9.4 (3.0)	11.6 (3.6)	11.8 (4.1)	0.9 (− 1.7 to 3.5)
**Pain (0–10)**	4.8 (2.2)	4.4 (3.0)	5.0 (3.0)	3.0 (2.2)	1.4 (− 0.3 to 3.2)
**SF-36 (10–30)**	18.7 (5.0)	19.5 (4.3)	21.2 (4.3)	21.5 (4.4)	− 0.6 (− 4.8 to 3.5)
**EQ5D VAS (0–100)**	62.3 (26.0)	75.3 (16.7)	65.6 (20.4)	70.6 (25.1)	3.5 (− 21.1 to 28.2)
**COPM performance (1–10)**	5.1 (2.6)	7.1 (2.6)	3.6 (2.0)	6.9 (3.2)	− 1.7 (− 4.1 to 0.7)
**COPM satisfaction (1–10)**	5.1 (3.3)	7.4 (3.0)	4.6 (3.0)	7.4 (3.4)	− 0.9 (− 3.7 to 1.9)

Mean between groups difference = [(t1_Nteleheabilitation_ − baseline_Ntelerehabilitation_)/17] − [(t1_Ncontrol_ − baseline_Ncontrol_)/17]

*TUG* Timed Up and Go, *30CST* 30-s Chair Stand Test, *SF-36* Medical Outcomes Short-Form Health Survey, *EQ-5D-3L VAS* EuroQol-5D Visual Analogue Scale, *COPM* Canadian Occupational Performance Measure, *t1* start of the outpatient physiotherapy, *SD* Standard deviation, *CI* Confidence interval

### Sample size estimation for the definitive trial

The sample size estimation resulted in a minimum of 140 participants per group for the definitive trial.

## Discussion

This study evaluated the feasibility of a telerehabilitation program using a smartphone app for older adults waiting for outpatient physiotherapy after hospital discharge. All criteria to proceed with the definitive study were accomplished.

The recruitment rate and satisfaction with the telerehabilitation program were better than expected, considering that data collection occurred during the COVID-19 pandemic. A reasonable number of older adults were recruited and only 8% of potentially eligible older adults refused to participate. Allowing older adults who did not own a smartphone but had a companion who did own one facilitated the participation of older adults who were unfamiliar with technology. The good acceptability to participate may be due to the interest in immediate assistance compared with the long waiting period for outpatient physiotherapy. The emerging use of technologies during the COVID-19 pandemic [51] may have also influenced the acceptability since the population has been adapting to receiving remote health care. These factors may explain the high satisfaction (9.7/10) of participants with the telerehabilitation program. Therefore, criteria related to recruitment rate and satisfaction to proceed with the definitive study were reached.

The dropout rate was lower than expected, and the telerehabilitation program was safe. Participants who dropped out of the study reported reasons unrelated to the program. One participant reported poor internet quality, hampering access to the app. Internet quality is challenging for remote interventions and has affected the complete adherence of four participants to this program. Two participants recorded the exercises and registered in the app when internet was available, and two stopped performing the exercises due to lack of internet. Despite the emerging use of internet due to the COVID-19 pandemic worldwide, its unavailability is still a problem [52, 53]. No serious adverse events were associated with the telerehabilitation program. Also, participants who performed the exercises alone reported no serious adverse events, suggesting that the telerehabilitation program was safe even without a companion. The perceived safety score was high, revealing that participants considered the program safe. Therefore, considering the dropout rate of less than 15% and no serious adverse events, these criteria were also reached to proceed with the definitive study.

The mean adherence to the telerehabilitation program was higher than twice a week, and most participants adhered to twice weekly sessions for at least 69% of the weeks spent waiting for outpatient physiotherapy. These data corroborate the literature, which reported that older adults performed the proposed exercises 1.5 to 3 times per week with an adherence rate between 58 and 77% [54]. We expected low adherence due to sample characteristics: older adults after hospital discharge and depending on internet access to perform the program. Older adults must be motivated to manage exercise performance at least twice a week since telerehabilitation programs have no professional supervision at home [55]. Thus, strategies to encourage adherence were adopted (e.g., frequent phone calls or in-person contact, positive feedback, reinforcement of exercise importance, and messages from the app) [29]. Other factors that prevented the complete adherence were unrelated to the program. In these cases, the companion was essential to manage the app. Despite instructions to only perform the exercises in the presence of a companion, some participants performed the exercise program alone. The unavailability of a companion was the most common reason for exclusion during screening. Thus, more participants could have been included in the study if this criterion was mandatory only for older adults not familiar with their smartphones. To ensure the safety of the intervention, we did not increase exercise intensity if the companion was unavailable during exercise performance. The adherence criterion to proceed with the definitive study was reached, and we changed the protocol to participants to perform the exercises alone, but they still had a companion for study eligibility.

Preliminary clinical outcomes suggest a trend towards maintaining conditioning during the waiting period in both the TG and CG. There was a decrease of 3 s in the TUG test for the TG and a reduction of nearly 1 s in the CG. Considering that a reduction between 0.8 and 3.1 s is clinically relevant for older adults [39, 40], our preliminary results suggest an increased agility in both groups. Participants in the TG remained in the waiting list for a longer period compared to those in the CG. This difference could be attributed to the small sample size of this feasibility study, since randomization typically ensures similarity between groups in larger samples. However, the results regarding clinical outcomes should be considered with caution since this was a feasibility study. The effectiveness of the telerehabilitation program will be tested with a larger sample using a model to control for waiting time to avoid biased results [29].

One of the strengths of this study is the establishment of criteria to proceed with the definitive study. These criteria allowed for a more precise decision-making regarding the continuity of the clinical trial and to identify potential challenges that could compromise its execution. This contributes to the overall success of the main study. Additionally, this study contributes to the scientific community by addressing the use of telerehabilitation in the context of public health in developing countries, such as Brazil.

The main limitation of this study was the recruitment during the COVID-19 pandemic. The pandemic resulted in a shorter waiting time for outpatient physiotherapy than expected (approximately three months prior to the pandemic). This was primarily due to the decreased number of elective surgeries and reduced service demand during that time*.* Therefore, we cannot predict the study results for a longer waiting list. Although participants had different diseases, most had musculoskeletal conditions, as commonly observed in rehabilitation centers. Additionally, our study employed a predefined strategy for estimating sample size [49, 50], which may not accurately represent the necessary sample size for some of our feasibility variables [56]. Furthermore, the estimated sample size for the definitive study should be treated with caution, as it was estimated using effect sizes from a small sample, which may not accurately reflect the variation found in larger samples. Finally, the waiting period was different for each participant, possibly influencing adherence and clinical outcomes. These factors will be verified with a larger sample in the definitive study.

## Conclusions

The present study demonstrated that the multicomponent exercises program using telerehabilitation with smartphone app for older adults on the waiting list for outpatient physiotherapy after hospital discharge was safe and presented high participants’ satisfaction and adequate adherence, recruitment, and dropout rates. The telerehabilitation program was feasible, and the definitive study can be conducted with few modifications.

### Supplementary Information

Additional file 1: CONSORT checklist.

Additional file 2: Sample size calculation.

## Data Availability

The datasets used during the current study are available from the corresponding author on reasonable request.

## Abbreviations

30CST: 30-S Chair Stand Test
CG: Control group
COPM: Canadian Occupational Performance Measure
EQ-5D-3L: EuroQol-5D
SF-36: Medical Outcomes Short-Form Health Survey
TG: Telerehabilitation group
TUG: Timed Up and Go
