# Supplementary figures and images for: App-based telerehabilitation program for older adults on waiting list for physiotherapy after hospital discharge: a feasibility pragmatic randomized trial
Source: Pilot Feasibility Stud. 2024 Jul 3;10:98. doi: 10.1186/s40814-024-01521-4 (PMC11221181; doi:10.1186/s40814-024-01521-4)

**Additional file 2. Sample size calculation for the definitive trial using G*Power software**


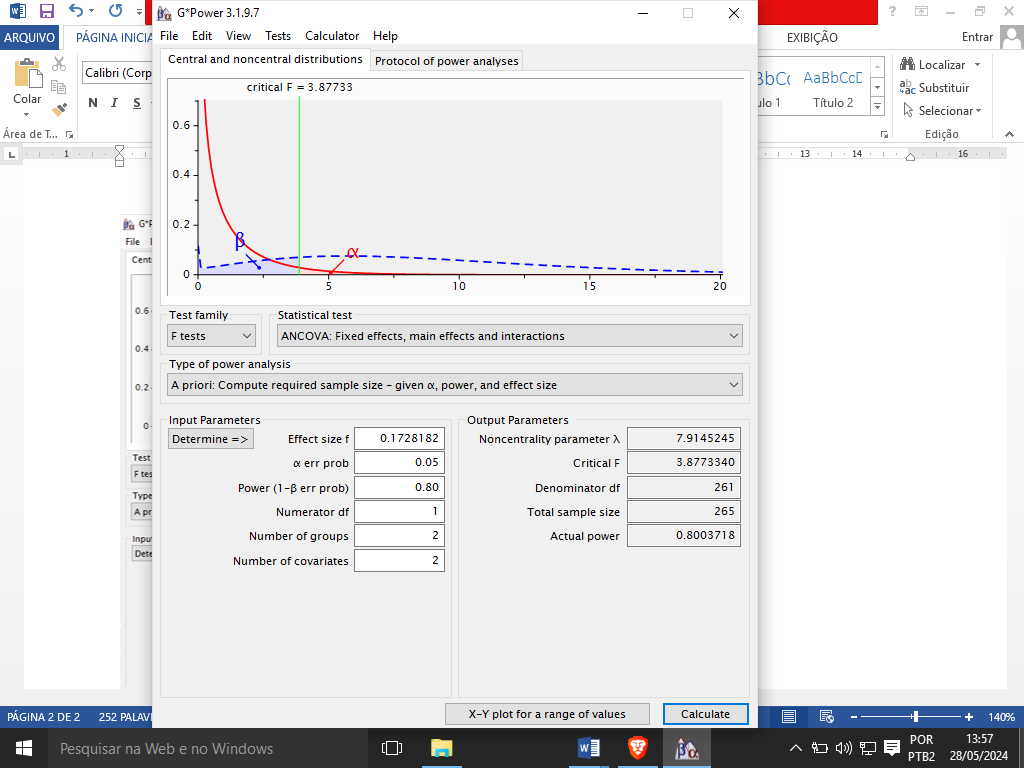

Supplement: Supplementary file 2 — Additional file 2: Sample size calculation. [file 40814_2024_1521_MOESM2_ESM.docx]
